# Predicting the daily gastrointestinal doses of stereotactic body radiation therapy for pancreatic cancer based on the shortest distance between the tumor and the gastrointestinal tract using daily computed tomography images

**DOI:** 10.1259/bjro.20230043

**Published:** 2023-10-18

**Authors:** Yusuke Uchinami, Takahiro Kanehira, Keiji Nakazato, Yoshihiro Fujita, Fuki Koizumi, Shuhei Takahashi, Manami Otsuka, Koichi Yasuda, Hiroshi Taguchi, Kentaro Nishioka, Naoki Miyamoto, Kohei Yokokawa, Ryusuke Suzuki, Keiji Kobashi, Keita Takahashi, Norio Katoh, Hidefumi Aoyama

**Affiliations:** 1 Department of Radiation Oncology, Hokkaido University Faculty of Medicine and Graduate School of Medicine, Sapporo, Japan; 2 Department of Medical Physics, Hokkaido University Hospital, Sapporo, Japan; 3 Department of Radiation Oncology, Hokkaido University Hospital, Sapporo, Japan; 4 Global Center for Biomedical Science and Engineering, Hokkaido University Faculty of Medicine, Sapporo, Japan; 5 Institute of Health Science Innovation for Medical Care, Hokkaido University Hospital, Sapporo, Japan

## Abstract

**Objectives:**

We aimed to investigate whether daily computed tomography (CT) images could predict the daily gastroduodenal, small intestine, and large intestine doses of stereotactic body radiation therapy (SBRT) for pancreatic cancer based on the shortest distance between the gross tumor volume (GTV) and gastrointestinal (GI) tract.

**Methods:**

Twelve patients with pancreatic cancer received SBRT of 40 Gy in five fractions. We recalculated the reference clinical SBRT plan (PLAN_ref_) using daily CT images and calculated the shortest distance from the GTV to each GI tract. The maximum dose delivered to 0.5 cc (D_0.5cc_) was evaluated for each planning at-risk volume of the GI tract. Spearman’s correlation test was used to determine the association between the daily change in the shortest distance (Δshortest distance) and the ratio of ΔD_0.5cc_ dose to D_0.5cc_ dose in PLAN_ref_ (ΔD_0.5cc_/PLAN_ref_) for quantitative analysis.

**Results:**

The median shortest distance in PLAN_ref_ was 0 mm in the gastroduodenum (interquartile range, 0–2.7), 16.7 mm in the small intestine (10.0–23.7), and 16.7 mm in the large intestine (8.3–28.1 mm). The D_0.5cc_ of PLAN_ref_ in the gastroduodenum was >30 Gy in all patients, with 10 (83.3%) having the highest dose. A significant association was found between the Δshortest distance and ΔD_0.5cc_/ PLAN_ref_ in the small or large intestine (*p* < 0.001) but not in the gastroduodenum (*p* = 0.404).

**Conclusions:**

The gastroduodenum had a higher D_0.5cc_ and predicting the daily dose was difficult. Daily dose calculations of the GI tract are recommended for safe SBRT.

**Advances in knowledge:**

This study aimed to predict the daily doses in SBRT for pancreatic cancer from the shortest distance between the GTV and the gastrointestinal tract.

Daily changes in the shortest distance can predict the daily dose to the small or large intestines, but not to the gastroduodenum.

## Introduction

Stereotactic body radiation therapy (SBRT) for pancreatic cancer is used to improve treatment outcomes and has been widely adopted.^1–3^ The advantages of SBRT include delivery of higher doses to the tumor and a shorter treatment period, which shortens the interruption of intense systemic chemotherapy. However, higher radiation dose delivery to the target lesion is often limited by the proximity of radiosensitive gastrointestinal (GI) organs. Moreover, inter- or intrafractional motion occurs in the GI tract,^4–6^ causing unexpectedly high radiation doses to be delivered to the GI tract.^7–9^ Therefore, daily dose evaluation is essential to ensure the safe delivery of SBRT.

Online adaptive radiotherapy has been used to address these challenges, and several therapeutic outcomes assessed using magnetic resonance-guided linear accelerators (MR-LINAC) or artificial intelligence-driven systems, such as ETHOS (Varian Medical Systems, Palo Alto, CA), have been reported in patients with pancreatic cancer.^10,11^ However, the availability of adaptive therapy is limited as it requires specialized equipment and qualified healthcare professionals (such as medical physicists or physicians). Moreover, dose uncertainties due to intrafractional motion may occur as the image acquisition to beam-on process may take several tens of minutes when using MR-LINAC or ETHOS.^10,12^

Among the online adaptive workflow steps before beam delivery, contouring is the most time-consuming step.^13,14^ However, daily plan adaptation is no longer necessary in >40% of total fractions.^14^ Therefore, it may be useful to quickly determine a treatment plan based on the shortest distance between the gross tumor volume (GTV) and organs at risk (OAR) measured using cone-beam computed tomography (CBCT) images.

Determining the distance between the tumor and the GI tract is essential to achieve dose constraints for OAR and to identify patients who may benefit from adapting the treatment plan.^14^ In this study, we focused on the shortest distance from the GTV to the GI tract and presumed that the daily GI tract dose could be predicted according to its distance. We aimed to investigate whether daily CT can predict the daily GI tract doses based on the shortest distance between the tumor and the GI tract.

## Methods and Materials

### Patients

This study was approved by the relevant institutional Ethics Review Committee (IRB number: 022–0193). All patients provided written informed consent for treatment. We retrospectively reviewed the records of patients treated with SBRT for pancreatic cancer between September 2020 and April 2023. Patients whose CT images were obtained prior to beam delivery on each treatment day, who underwent SBRT using intensity-modulated radiotherapy (IMRT), and who had a tumor located in the pancreatic head or body were included. Finally, the data of 12 patients who met these criteria were analyzed. Table 1 shows the patients’ characteristics.

**Table 1.** Patients’ background characteristics

Patients (*n* = 12)	
Male/female	8/4
Age (median)	80 years (range: 71–89)
Pancreatic head/body	6/6
GTV volume (median)	6.9 cm^3^ (IQR: 3.9–14.7)

GTV, gross tumor volume; IQR, interquartile range; SBRT, stereotactic body radiation therapy

For respiratory-gated SBRT using a fiducial marker, a metallic embolization coil was implanted transarterially or gold anchors were implanted in the retroperitoneal region near the tumor in all patients. A dose was delivered when a marker was within ±2.0 mm of the planned coordinates relative to the isocenter (at natural expiration).^15^ SBRT was delivered with six mega-voltage (MV) beams with or without flattening filter-free by a TrueBeam (Varian Medical Systems), while implementing a SyncTraX FX4 (Shimadzu, Kyoto, Japan) for fiducial marker-based respiratory gating. At patient set-up, CBCT images were obtained and bone matching was performed using a six-dimensional couch. Translation-only registration was then performed based on the fiducial marker position. The protocol from marker implantation to SBRT was similar to that reported previously concerning lung or liver cancer.^16,17^ The SBRT plan was generated using step-and-shoot IMRT of 7–10 portals and planned using Pinnacle^3^ version 14.0 (Philips, Amsterdam, Netherlands) software.

### Planning CT scan

CT images were obtained after fasting for at least 6 h. Four-dimensional CT (4DCT) was performed using a real-time position management system (Varian Medical Systems, Palo Alto, CA, USA). The acquired 4DCT images were divided into 10 different three-dimensional (3D) CT images according to time-based sorting in 10 respiratory phases (0%, 10%, ..., 90%). Here, 0% CT implies CT performed at natural inspiration, while 50% CT implies CT performed at natural expiration. After the 4DCT scan, enhanced or non-enhanced planning CT was performed with a slice thickness of 2 mm at natural expiration as respiratory gating was carried out during this respiratory phase. During the planning CT scan, the CT images were examined to determine if they had been correctly obtained at natural expiration, which refers to the 50% phase of the 4DCT images. In this case, the 4DCT images were used only to confirm natural expiration and not used for the treatment planning.

### SBRT planning

The treatment plan was generated on CT images at natural expiration, and available contrast-enhanced CT was registered against the planning CT for accurate delineation. The GTV was defined according to the available imaging data, including those obtained using diagnostic enhanced/non-enhanced CT, magnetic resonance imaging (MRI), and positron emission tomography. The clinical target volume (CTV) was generated as the GTV along with the tumor-vessel interface (TVI), which was defined as areas of major blood vessels within the GTV with a 5 mm margin.^18^ The planning target volume (PTV) was created through adding a 5 mm margin to the CTV. The planning at-risk volume (PRV) was generated through adding a 5 mm margin to each gastrointestinal organ, including the stomach, duodenum, small intestine, and large intestine (stomach_PRV, duodenum_PRV, small intestine_PRV, and large intestine_PRV, respectively). These organs were contoured according to Radiation Therapy Oncology Group consensus guidelines.^19^ The modified PTV (PTV_mod_) was defined as the PTV minus the area overlapping the GI tract_PRV. The goal of the dose prescription was 40 Gy (100% of the prescribed dose) in five fractions for 90% of the PTV_mod_ surrounded by 80–90% isodose lines.^18^

The OAR dose constraint for each PRV was the volume receiving 38 Gy (V_38_) <0.5 cm^3^ until January 2021 (patients 1–2) or V_33_ <0.5 cm^3^ (patients 3–12) thereafter. The other primary target goals and dose constraints are listed in Table 2. The initial SBRT plan was referred to as the reference plan (PLAN_ref_).

**Table 2.** General dose constraints for SBRT

Contour	Dose constraints	
GTV	D_50%_ ≥ 40 Gy, (acceptable, D_2%_ ≥ 40 Gy)	
PTV_mod_	D_90%_ ≥ 40 Gy, (acceptable, D_90%_ ≥ 32 Gy)	
PRVs of the GI tract	until Jan 2021	from Feb 2021
Stomach_PRV	V_38_<0.5 cm^3^	V_33_<0.5 cm^3^
Duodenum_PRV	V_38_<0.5 cm^3^	V_33_<0.5 cm^3^
Small intestine_PRV	V_38_<0.5 cm^3^	V_33_<0.5 cm^3^
Large intetsinte_PRV	V_38_<0.5 cm^3^	V_33_<0.5 cm^3^
Kidneys, bilateral	V_12_<25%	
Liver	V_12_<40%	
Spinal cord + 5 mm	V_20_<0.5 cm^3^	

GI tract, gastrointestinal tract; GTV, gross tumor volume; PRV, planning at risk volume; PTV_mod_, planning target volume for evaluation; SBRT, stereotactic body radiation therapy.

V_X_ refers to volumes receiving X Gy at least; D_X%_ refers to the dose received by X percent of the target volume at least

### Dose Evaluation

A non-contrast-enhanced CT (CT_eval_) for daily dose evaluation was performed before each SBRT fraction under the same conditions as the treatment planning CT. These CT images were scanned with a treatment planning CT system following the same CT imaging conditions as the treatment planning CT scan in terms of CT equipment, parameters for CT imaging, vacuum cushion, body position, dietary restrictions, and respiratory status (natural expiration). For dose evaluation, the contours of the target and risk organs were generated as in the PLAN_ref_ and were reviewed by at least two radiation oncologists. Here, the contours were manually created on the daily CT because the quality of the contours was not satisfactory in terms of deformable image registration, especially in the GI tract. The dose was recalculated on CT_eval_ using the original beam data from the PLAN_ref_. The recalculated plan for the daily dose evaluation was referred to as the PLAN_eval_. Prior to the daily dose evaluation, the 3D coordinates of the isocenter in the PLAN_eval_ were determined based on the relationship between the 3D coordinates of the fiducial marker and those of the isocenter in the PLAN_ref_. These methods are similar to those reported in previous studies regarding interfractional motion.^7^ Five fractions were evaluated for each of the 12 patients, resulting in 60 PLAN_evals_.

### Data Analysis

To determine the locational relationship, the 3D shortest distance between the surface of the GTV and the GI tract was calculated using MIM Maestro software version 7.0 (MIM Software, Cleveland, OH, USA). In the exploratory data analysis, differences in the boundary between the stomach and the duodenum could potentially affect the calculated shortest distance from the GTV to the stomach or duodenum. In some cases, for example, a larger contour of the stomach may result in a shorter distance to the tumor, whereas a smaller contour may result in a longer distance to the tumor. These changes are due to differences in contours rather than interfractional motion of the GI tract. Therefore, the stomach and duodenum were evaluated together as the gastroduodenum.^20^

We aimed to determine whether the shortest distance between the GTV and the GI tract could predict the daily dose delivered to the GI tract. Therefore, we evaluated the association between daily changes in the shortest distance and daily doses. For dose evaluation, the D_0.5cc_ of PRV was extracted from the PLAN_ref_ and PLAN_eval_. D_Xcc_ was defined as the maximum dose delivered to a volume of X cc to the organ of interest. The dosimetric parameters and shortest distance were calculated using the following formulae to quantify the daily change:

ΔShortest distance=shortest distance_PLANeval−shortest distance_PLANref

ΔDxcc=Dxcc_PLANeval−Dxcc_PLANref

Since the dose constraints of the GI tract differed between patients, ratios were used for quantitative analysis of the daily dose changes (ΔD_Xcc_). We examined the association between the Δshortest distance and the ΔD_Xcc_/ PLAN_ref_, which represents the dose ratio of ΔD_Xcc_ to D_Xcc_ of the PLAN_ref_. Spearman’s correlation test was used in the statistical analysis to determine the association between the Δshortest distance and the ΔD_Xcc_/ PLAN_ref_.

The paired differences were analyzed using paired Wilcoxon signed-rank tests, and categorical variables were analyzed using a chi-square test. For comparison of more than two groups, Kruskal–Wallis tests were used with post-hoc Dunn’s tests. The coefficient of variation, calculated as the standard deviation (SD) divided by the mean, was also evaluated. A p-value < 0.05 was considered significant, and JMP Pro version 16.0 (SAS, Cary, NC) software was used to perform all statistical analyses.

## Results

### Shortest Distance

**Figure 1.**
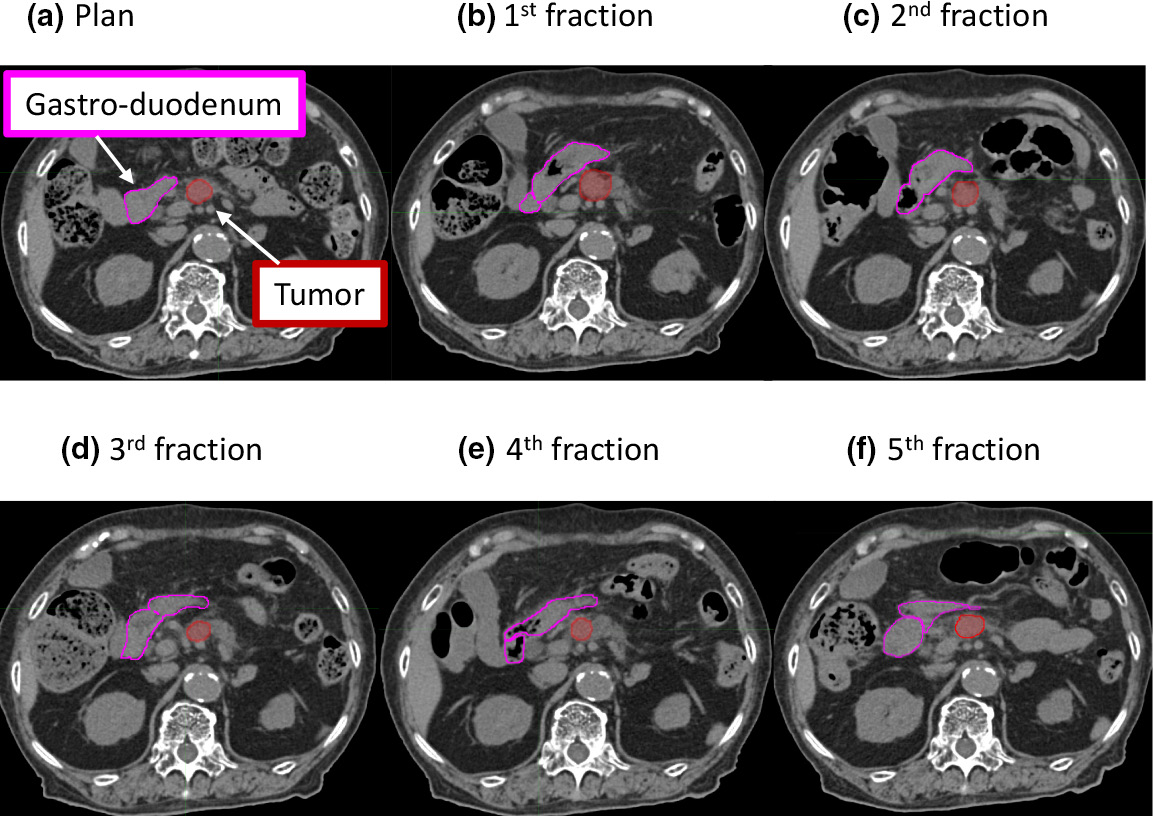
A case showing interfractional motion The gross tumor volume (red) and gastroduodenum (pink) are shown on each CT image. The shape of the gastrointestinal tract is different due to the interfractional motion, leading to daily changes in the shortest distance between the tumor and gastrointestinal tract. CT, computed tomography.

**Figure 2.**
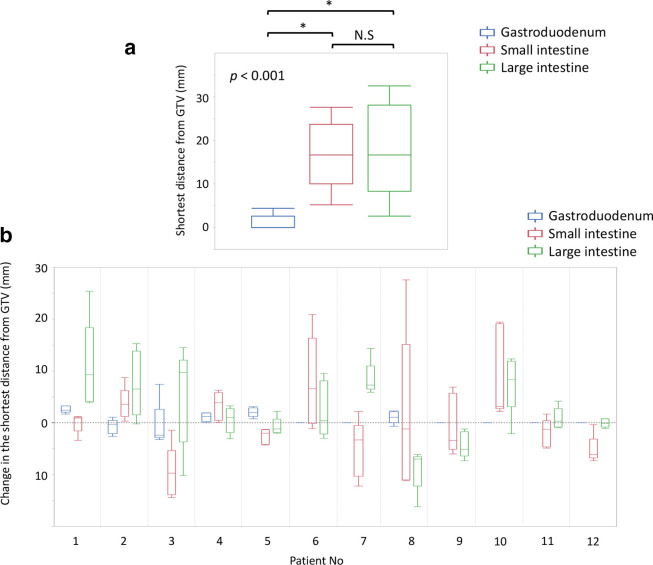
(a) The shortest distance from the GTV to the GI tract in the PLAN_ref_ and (b) daily changes in the shortest distance in each patient. (a) * indicates a significance between the two groups using Dunn’s test. (b) The change in the shortest distance to the gastroduodenum in patients 6, 7, 9–12 is zero, which means the GTV and gastroduodenum are always adjacent to each other in the planning CT and daily CT images. GI tract, gastrointestinal tract; GTV, gross tumor volume; NS, not significant.

In the PLAN_ref_ and PLAN_eval_ review, interfractional motion was observed even if the CT images were obtained at natural expiration and after fasting for at least 6 h (Figure 1). The median shortest distances between the GTV and the GI tract in the PLAN_ref_ were 0 mm (IQR, 0–2.7) in the gastroduodenum, 16.7 mm (10.0–23.7) in the small intestine, and 16.7 mm (8.3–28.1) in the large intestine (Figure 2a). The distance was significantly smaller in the gastroduodenum than that in the small or large intestines (*p* < 0.001). The mean coefficients of variation ±SD in the shortest distance were 0.07 ± 0.34 in the gastroduodenum, 0.31 ± 0.20 in the small intestine, and 0.20 ± 0.13 in the large intestine.

The mean Δshortest distances with SD were 0.5 ± 1.7 mm in the gastroduodenum, 0 ± 8.0 mm in the small intestine, and 2.5 ± 7.2 mm in the large intestine (Figure 2b). The median absolute values of the Δshortest distance were 0 mm (IQR, 0–2.0) in the gastroduodenum, 3.4 mm (1.2–8.1) in the small intestine, and 4.3 mm (1.2–8.3) in the large intestine (*p* < 0.001).

### Dose Evaluation

**Figure 3.**
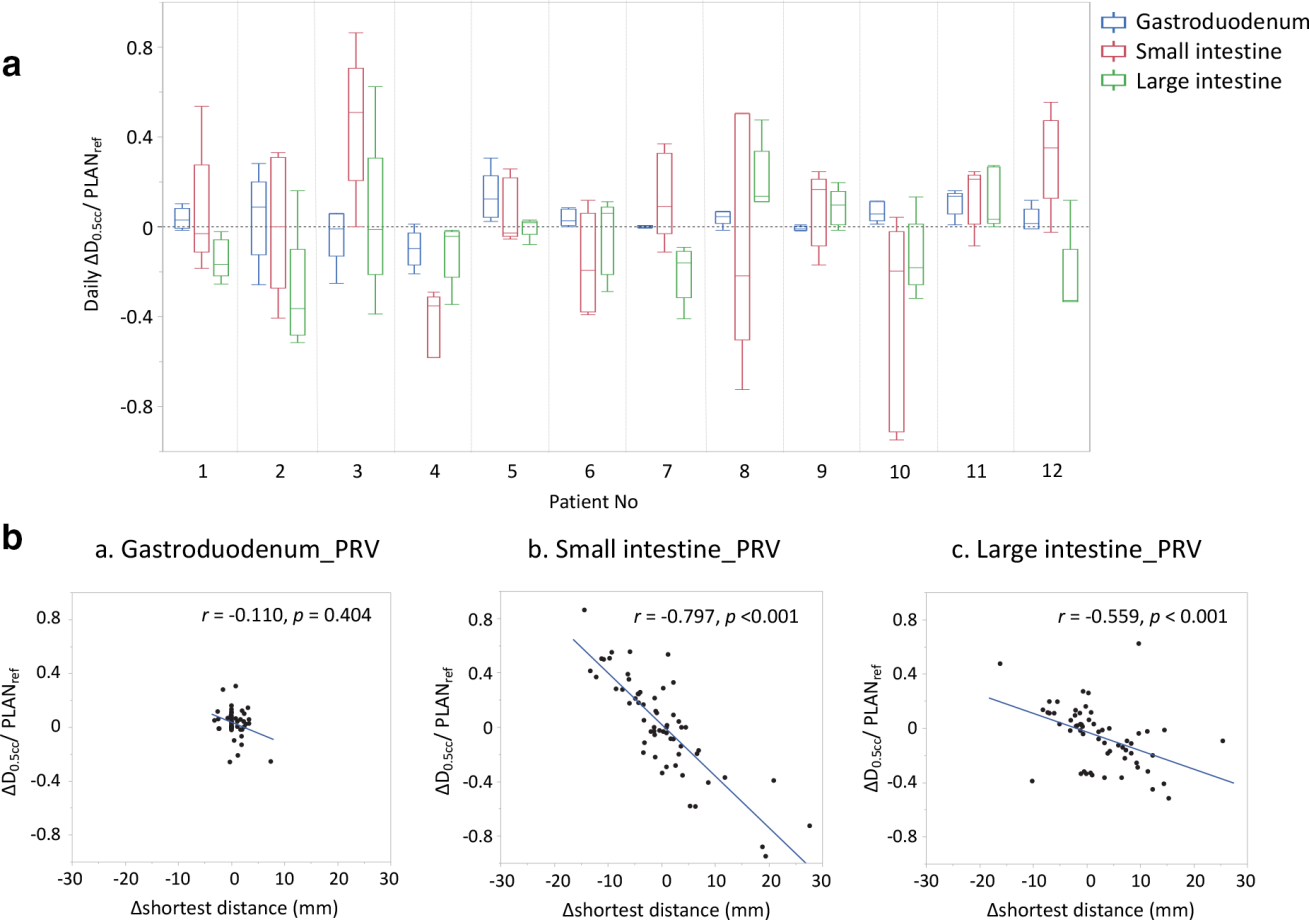
(a) The ΔD_0.5cc_/ PLAN_ref_ in each patient and the (b) association between the Δshortest distance and ΔD_0.5cc_/ PLAN_ref_. (a) ΔD_0.5cc_/ PLAN_ref_ indicates the ratio of ΔD_0.5cc_ dose to D_0.5cc_ dose in PLAN_ref_ for quantitative analysis (b) The horizontal axis shows the Δshortest distance, while the vertical axis shows the ΔD_0.5cc_/ PLAN_ref_ of PRV. The single dot indicates the evaluation of one fraction (PLAN_eval_) in a case. As 12 patients were treated with radiotherapy delivered in five fractions, each panel contains a total of 60 dots. The (r) in each panel indicates the Spearman’s correlation coefficients. GI tract_PRV, planning at-risk volume of the gastrointestinal tract; GTV, gross tumor volume.

In the PLAN_ref_, the D_0.5cc_ was >30 Gy in the gastroduodenum_PRV (Table 3). Ten of 12 patients (83.3%) had the highest D_0.5cc_ in the gastroduodenum. The mean ΔD_0.5cc_/ PLAN_ref_ ±SD values were 0.03 ± 0.10 in the gastroduodenum_PRV, 0.02 ± 0.36 in the small intestine_PRV, and −0.06 ± 0.22 in the large intestine_PRV (Figure 3a). In Spearman’s correlation test between the Δshortest distance and ΔD_0.5cc_/ PLAN_ref_, a significant association was found in the small intestine (correlation coefficient (*r*) = −0.797, *p* < 0.001) and large intestine (*r* = −0.559, *p* < 0.001), but not in the gastroduodenum (*r* = −0.110, *p* = 0.404; Figure 3b). Similar correlations were obtained for other dose levels (D_1cc_, D_5cc_, and D_10cc_) (Table 4).

**Table 3.** D_0.5cc_ (Gy) at PLAN_ref_

Patient	Tumor location in the pancreas	Gastroduodenum		Small intestine		Large intestine	
		Organ itself	PRV	Organ itself	PRV	Organ itself	PRV
1	Body	27.8	37.4	17.4	19.4	16.2	18.5
2	Body	25.7	33.8	22.4	33.0	20.6	30.3
3	Body	26.4	32.4	15.2	18.3	15.7	16.0
4	Body	24.2	31.8	15.7	29.2	11.8	12.2
5	Body	27.4	32.2	27.6	32.6	25.9	29.5
6	Head	25.8	30.8	26.8	32.0	22.8	30.0
7	Head	30.8	33.0	14.3	23.0	23.7	28.2
8	Head	26.8	31.8	11.3	19.2	16.3	19.7
9	Body	30.5	32.4	21.1	28.2	15.9	18.8
10	Head	31.3	32.9	9.9	11.6	26.1	30.6
11	Head	31.6	32.7	25.5	32.1	18,1	25.3
12	Head	30.8	34.9^*a*^	9.7	13.3	19.0	29.6

PLAN_ref_, the initial stereotactic body radiation therapy reference plan; PRV, planning at risk volume.

The critical dose constraint in the GI tract was V_38_ < 0.5 cm^3^ (patients 1 and 2) or V_33_<0.5 cm^3^ (patients 3–12).

D_0.5cc_ refers to the maximum dose delivered to a volume of 0.5 cc in each organ.

a For patient No.12, considering the dose to the tumor, the gastroduodenal dose in the PLAN_ref_ was deemed clinically acceptable.

**Table 4.** Spearman’s correlation test between the Δshortest distance and the daily dose

Parameter	ΔD_1cc_/ PLAN_ref_		ΔD_5cc_/ PLAN_ref_		ΔD_10cc_/ PLAN_ref_	
	CC	*p*-value	CC	*p*-value	CC	*p*-value
Gastroduodenum_PRV	-0.101	0.441	-0.003	0.980	0.043	0.743
Small intestine_PRV	-0.807	<0.001	-0.788	<0.001	-0.716	<0.001
Large intestine_PRV	-0.610	<0.001	-0.577	<0.001	-0.516	<0.001

CC, correlation coefficients; PLAN_ref_, reference plan of stereotactic body radiotherapy for pancreatic cancer; PRV, planning at risk volume.

ΔD_Xcc_/PLAN_ref_ means the ratio of ΔD_Xcc_ dose to D_Xcc_ dose in PLAN_ref_

## Discussion

This study investigated the dosimetric parameters of SBRT for pancreatic cancer in terms of the locational relationship between the tumor and GI tract. We quantified daily changes in the shortest distance from the GTV to the GI tract and their effect on daily GI tract doses. The distance was significantly shorter in the gastroduodenum than in the small or large intestine; therefore, the gastroduodenum would be more likely to receive high radiation doses. Spearman’s correlation test results showed that daily GI tract doses could be predicted using the shortest distance in the small or large intestine, but not in the gastroduodenum, possibly due to its proximity to the GTV. Hence, dose recalculation is essential for predicting daily doses as determination of the gastroduodenal dose is needed to ensure safe delivery of SBRT for pancreatic cancer.

One challenge associated with SBRT is limiting GI tract doses to reduce the risk of late adverse events. Several studies have investigated the association between dosimetric parameters and late adverse events in the GI tract. In relation to the stomach, Feng et al proposed a normal tissue complication probability (NTCP) model to predict the risk of radiation-induced gastric bleeding.^21^ They reported that the highest dose was a critical predictor rather than the mean dose in the stomach. In relation to the duodenum, the maximum or D_1cc_ dose was reported to be more critical in predicting late toxicities.^22,23^ Based on these studies, the delivery of a high radiation dose to a small volume of the stomach or duodenum is closely associated with the occurrence of late adverse events; hence, dose reduction is necessary. In our study, D_0.5cc_ was used in the dose analysis as the institutional dose constraint was V_33_ or V_38_ < 0.5 cm^3^ for PRV. In our study, we analyzed the stomach and the duodenum as one organ; therefore, no conclusions can be drawn for the stomach or duodenum separately. Nonetheless, the D_0.5cc_ dose was significantly higher in the gastroduodenum than that in the small or large intestine owing to its proximity to the GTV. The risk of developing late adverse events is likely to be higher in the gastroduodenum, as suggested in previous studies.^24,25^

Regarding the association between the Δshortest distance and ΔGI tract dose, a proportional dose-distance relationship was found in the small intestine and large intestine, but not in the gastroduodenum, probably due to differences in the shortest distance in each GI organ. In the sections of the GI tract located near the tumor, such as the gastroduodenum, an SBRT plan is highly optimized using steep dose distribution curves to ensure adherence to the dose constraints of the GI tract. However, the PLAN_eval_ involved dose distribution obtained by recalculating the original beam data of the PLAN_ref_, which indicated that the PLAN_eval_ was not precisely optimized based on daily CT images. The shortest distance between the gastroduodenum and the GTV in the PLAN_eval_ was ≤6 mm in all patients (100%). Therefore, a slight positional change due to interfractional motion can lead to dose uncertainty in terms of predictability in the PLAN_eval_.

In contrast, non-highly optimized areas such as the small or large intestine have gentle and linear dose distribution curves. We found that the shortest distance in most of the PLAN_eval_ was ≥6 mm in the small and large intestine. This background may explain the proportional association between the Δshortest distance and daily dose differences (ΔD_0.5cc_/ PLAN_ref_) in the small and large intestine (Figure 3b). Similar results were reported by Loi et al, who examined 35 patients with pancreatic cancer treated with SBRT and compared the planned and daily values of the dosimetric parameters, with the daily value of D_2cc_ being significantly higher in the stomach or duodenum but not in the bowel.^8^ Their study did not consider the distance between the GTV and OAR; however, daily dose uncertainties might be prominent in the stomach or duodenum.

Our study findings suggest that prediction of the daily dose in the gastroduodenum is difficult when based only on the shortest distance between the GTV and GI tract using daily CT images. Our initial hypothesis was that a shorter distance would result in a higher D_0.5cc_, while a longer distance would result in a lower D_0.5cc_. As expected, the daily dose-distance relationship was proportional in relation to the small and large intestines. Contrastingly, the short distance between the gastroduodenum and GTV in pancreatic cancer may make it difficult to predict daily dose changes due to interfractional motion. Considering that 10 of 12 patients received the highest D_0.5cc_ in the gastroduodenum and that its dose is important to predict the risk of late adverse events in the GI tract, daily dose calculation is essential to ensure the safe delivery of treatment.

This study had several limitations. First, the shortest distance was generally based on contours used in clinical practice. At least two radiation oncologists approved these distances for clinical SBRT planning; however, the possibility of slight variations cannot be excluded. In particular, the shortest distance from the GTV to the GI tract was measured in millimeters. Therefore, the effect of possible contouring error may not have been negligible. Moreover, since the slice thickness of CT images used in this study was 2 mm, it was difficult to contour the target and risk organs from CT images with a slice thickness of ≤1 mm. Another limitation is the small number of samples. We only included 60 sets of daily CT images from 12 patients in our analysis, which means that definitive conclusions cannot be drawn from this study; hence, future studies are needed involving more patients. Because of this limitation in terms of the small number of study participants, those with different dose constraints were analyzed together. A steeper dose gradient between the target and OAR was observed in the V_33_<0.5 cm^3^ group than in the V_38_<0.5 cm^3^ group. However, the ΔD_0.5cc_/ PLAN_ref_ of the gastroduodenum, which is the most important index in this study, did not differ significantly between patients with these two different dose constraints (*p* > 0.05).

In conclusion, the distance between the tumor and GI tract was smaller in the gastroduodenum than that in the small and large intestines. Prediction of the daily dose delivered to the small or large intestine may be possible based on the Δshortest distance using daily CT images; however, this method may be challenging to apply in the gastroduodenum. Recalculating the initial plan using daily CT images is essential to correctly evaluate the daily gastroduodenal dose.

## References

[1] Chuong MD, Springett GM, Freilich JM, Park CK, Weber JM, Mellon EA, et al . Stereotactic body radiation therapy for locally advanced and borderline Resectable Pancreatic cancer is effective and well tolerated. Int J Radiat Oncol Biol Phys 2013; 86: 516–22. doi: 10.1016/j.ijrobp.2013.02.022 23562768

[2] Herman JM, Chang DT, Goodman KA, Dholakia AS, Raman SP, Hacker-Prietz A, et al . Phase 2 multi-institutional trial evaluating Gemcitabine and stereotactic body radiotherapy for patients with locally advanced Unresectable Pancreatic adenocarcinoma. Cancer 2015; 121: 1128–37. doi: 10.1002/cncr.29161 25538019 PMC4368473

[3] Jumeau R, Delouya G, Roberge D, Donath D, Béliveau-Nadeau D, Campeau M-P . Stereotactic body radiotherapy (SBRT) for patients with locally advanced Pancreatic cancer: A single center experience. Dig Liver Dis 2018; 50: 396–400. doi: 10.1016/j.dld.2017.12.013 29326012

[4] Houweling AC, Fukata K, Kubota Y, Shimada H, Rasch CRN, Ohno T, et al . The impact of Interfractional anatomical changes on the accumulated dose in carbon ion therapy of Pancreatic cancer patients. Radiother Oncol 2016; 119: 319–25. doi: 10.1016/j.radonc.2016.03.004 26993417

[5] Houweling AC, Crama K, Visser J, Fukata K, Rasch CRN, Ohno T, et al . Comparing the Dosimetric impact of Interfractional anatomical changes in photon, proton and carbon ion radiotherapy for Pancreatic cancer patients. Phys Med Biol 2017; 62: 3051–3064. doi: 10.1088/1361-6560/aa6419 28252445

[6] Uchinami Y, Suzuki R, Katoh N, Taguchi H, Yasuda K, Miyamoto N, et al . Impact of organ motion on volumetric and Dosimetric parameters in stomach Lymphomas treated with intensity-modulated radiotherapy. J Appl Clin Med Phys 2019; 20: 78–86. doi: 10.1002/acm2.12681 PMC6698764 31400082

[7] Niedzielski JS, Liu Y, Ng SSW, Martin RM, Perles LA, Beddar S, et al . Dosimetric uncertainties resulting from Interfractional anatomic variations for patients receiving Pancreas stereotactic body radiation therapy and cone beam computed tomography image guidance. Int J Radiat Oncol Biol Phys 2021; 111: 1298–1309. doi: 10.1016/j.ijrobp.2021.08.002 34400267 PMC8651043

[8] Loi M, Magallon-Baro A, Suker M, van Eijck C, Sharma A, Hoogeman M, et al . Pancreatic cancer treated with SBRT: effect of anatomical Interfraction variations on dose to organs at risk. Radiother Oncol 2019; 134: 67–73. doi: 10.1016/j.radonc.2019.01.020 31005226

[9] Uchinami Y, Kanehira T, Fujita Y, Miyamoto N, Yokokawa K, Koizumi F, et al . Evaluation of short-term gastrointestinal motion and its impact on Dosimetric parameters in stereotactic body radiation therapy for Pancreatic cancer. Clin Transl Radiat Oncol 2023; 39: 100576. doi: 10.1016/j.ctro.2023.100576 36686564 PMC9852488

[10] Tyagi N, Liang J, Burleson S, Subashi E, Godoy Scripes P, Tringale KR, et al . Feasibility of Ablative stereotactic body radiation therapy of Pancreas cancer patients on a 1.5 Tesla magnetic resonance-Linac system using abdominal compression. Phys Imaging Radiat Oncol 2021; 19: 53–59. doi: 10.1016/j.phro.2021.07.006 34307919 PMC8295846

[11] Schiff JP, Price AT, Stowe HB, Laugeman E, Chin R-I, Hatscher C, et al . Simulated computed tomography-guided stereotactic adaptive radiotherapy (CT-STAR) for the treatment of locally advanced Pancreatic cancer. Radiother Oncol 2022; 175: 144–51. doi: 10.1016/j.radonc.2022.08.026 36063981

[12] Byrne M, Archibald-Heeren B, Hu Y, Teh A, Beserminji R, Cai E, et al . Varian ethos online adaptive radiotherapy for prostate cancer: early results of contouring accuracy, treatment plan quality, and treatment time. J Appl Clin Med Phys 2022; 23: e13479. doi: 10.1002/acm2.13479 34846098 PMC8803282

[13] Güngör G, Serbez İ, Temur B, Gür G, Kayalılar N, Mustafayev TZ, et al . Time analysis of online adaptive magnetic resonance-guided radiation therapy Workflow according to anatomical sites. Pract Radiat Oncol 2021; 11: e11–21. doi: 10.1016/j.prro.2020.07.003 32739438

[14] Bohoudi O, Bruynzeel AME, Meijerink MR, Senan S, Slotman BJ, Palacios MA, et al . Identification of patients with locally advanced Pancreatic cancer benefitting from plan adaptation in MR-guided radiation therapy. Radiother Oncol 2019; 132: 16–22. doi: 10.1016/j.radonc.2018.11.019 30825964

[15] Shirato H, Shimizu S, Kitamura K, Nishioka T, Kagei K, Hashimoto S, et al . Four-dimensional treatment planning and Fluoroscopic real-time tumor tracking radiotherapy for moving tumor. Int J Radiat Oncol Biol Phys 2000; 48: 435–442. doi: 10.1016/s0360-3016(00)00625-8 10974459

[16] Uchinami Y, Katoh N, Abo D, Taguchi H, Yasuda K, Nishioka K, et al . Treatment outcomes of stereotactic body radiation therapy using a real-time tumor-tracking radiotherapy system for hepatocellular Carcinomas. Hepatol Res 2021; 51: 870–879. doi: 10.1111/hepr.13649 33894086

[17] Inoue T, Katoh N, Onimaru R, Shimizu S, Tsuchiya K, Suzuki R, et al . Stereotactic body radiotherapy using Gated radiotherapy with real-time tumor-tracking for stage I non-small cell lung cancer. Radiat Oncol 2013; 8. doi: 10.1186/1748-717X-8-69 PMC3614446 23518013

[18] Oar A, Lee M, Le H, Hruby G, Dalfsen R, Pryor D, et al . Australasian gastrointestinal trials group (AGITG) and Trans-Tasman radiation oncology group (TROG) guidelines for Pancreatic stereotactic body radiation therapy (SBRT). Pract Radiat Oncol 2020; 10: e136–46. doi: 10.1016/j.prro.2019.07.018 31761541

[19] Jabbour SK, Hashem SA, Bosch W, Kim TK, Finkelstein SE, Anderson BM, et al . Upper abdominal normal organ contouring guidelines and Atlas: a radiation therapy oncology group consensus. Pract Radiat Oncol 2014; 4: 82–89. doi: 10.1016/j.prro.2013.06.004 24890348 PMC4285338

[20] Alam S, Veeraraghavan H, Tringale K, Amoateng E, Subashi E, Wu AJ, et al . Inter- and Intrafraction motion assessment and accumulated dose Quantification of upper gastrointestinal organs during magnetic resonance-guided Ablative radiation therapy of Pancreas patients. Phys Imaging Radiat Oncol 2022; 21: 54–61. doi: 10.1016/j.phro.2022.02.007 35243032 PMC8861831

[21] Feng M, Normolle D, Pan CC, Dawson LA, Amarnath S, Ensminger WD, et al . Dosimetric analysis of radiation-induced gastric bleeding. Int J Radiat Oncol Biol Phys 2012; 84: e1–e6. doi: 10.1016/j.ijrobp.2012.02.029 22541965 PMC3423508

[22] Kopek N, Holt MI, Hansen AT, Høyer M . Stereotactic body radiotherapy for Unresectable Cholangiocarcinoma. Radiother Oncol 2010; 94: 47–52. doi: 10.1016/j.radonc.2009.11.004 19963295

[23] Holyoake DLP, Aznar M, Mukherjee S, Partridge M, Hawkins MA . Modelling duodenum radiotherapy toxicity using cohort dose-volume-Histogram data. Radiother Oncol 2017; 123: 431–37. doi: 10.1016/j.radonc.2017.04.024 28600084 PMC5486774

[24] Jung J, Yoon SM, Park J, Seo D-W, Lee SS, Kim M-H, et al . Stereotactic body radiation therapy for locally advanced Pancreatic cancer. PLoS ONE 2019; 14: e0214970. doi: 10.1371/journal.pone.0214970 30978229 PMC6461258

[25] Courtney PT, Paravati AJ, Atwood TF, Raja N, Zimmerman CT, Fanta PT, et al . Phase I trial of stereotactic body radiation therapy dose escalation in Pancreatic cancer. Int J Radiat Oncol Biol Phys 2021; 110: 1003–12. doi: 10.1016/j.ijrobp.2021.02.008 33571625

